# A Survey of *Escherichia coli* and *Salmonella* in the Hyporheic Zone of a Subtropical Stream: Their Bacteriological, Physicochemical and Environmental Relationships

**DOI:** 10.1371/journal.pone.0129382

**Published:** 2015-06-12

**Authors:** Riccardo Mugnai, Ana Sattamini, José Augusto Albuquerque dos Santos, Adriana Hamond Regua-Mangia

**Affiliations:** 1 Laboratorio de Aracnologia, Departamento de Invertebrados Museu Nacional/Universidade Federal do Rio de Janeiro, Rio de Janeiro, Brazil; 2 Laboratório de Avaliação e Promoção da Saúde Ambiental, Fundação Oswaldo Cruz, Rio de Janeiro, Brazil; 3 Laboratório de Epidemiologia Molecular de Doenças Infecciosas/Departamento de Ciências Biológicas, Fundação Oswaldo Cruz, Rio de Janeiro, Brazil; Argonne National Laboratory, UNITED STATES

## Abstract

The Hyporheic Zone is among the most important interstitial freshwater habitats, but the relationship between biotic and abiotic factors in this zone remains under-explored. Enterobacteria were expected to be present, but no specific studies had ever confirmed this prediction. The aim of this study was, therefore, to evaluate the total coliforms, *Escherichia coli* and *Salmonella* spp. in hyporheic water and to determine the relationship of the physical, chemical and environmental factors at different depths in a rainforest stream. To this end, thirty-six water samples were collected at three depths in sites located in the first, second and third orders in diverse substrates. The total coliforms, *Escherichia coli* and *Salmonella* sp. were evaluated in terms of their CFU/ml. In the interstitial samples, coliforms were detected in 100% of the samples. The total coliform counts had higher values at intermediate depths, while *E*. *coli* and *Salmonella* spp. instead had higher values at intermediate and large depths, often reaching or exceeding the values of the surface samples. Our results revealed that *Salmonella* spp. and the coliforms have different microhabitat preferences. *Salmonella* spp. and coliform species prefer deposition areas, such as lateral sides of pools, curves and bars, but they have a tendency to distribute into different depths, likely due to temperature differences. *Salmonella* spp. prefer compact substrata, with fewer fluids passing through and with upwelling areas with lower oxygen inflow. The coliform species showed the opposite preference. Our results suggest that bacterial variation is related to environmental factors and physical-chemical parameters within the HZ and may play a key role in the microbial diversity and distribution in these ecosystems.

## Introduction

Many microorganisms are known to be relevant contaminants of the surface water of aquatic environments across diverse geographic areas [1,2,3,4,5]. As a means of assessing the level of fecal pollution in environmental waters, the use of enteric indicators, such as fecal coliforms (FC) or *Escherichia coli*, is widely accepted [6]. The validity of using such organisms as indicators of water pollution depends on their fecal specificity and inability to multiply outside their primary host environment, the gastrointestinal tracts of humans and warm-blooded animals [7]. Coliforms, *Escherichia coli* and *Salmonella* spp. reside in the gastrointestinal tracts of humans and animals and are used as indicator organisms to assess the microbiological safety of drinking and recreational waters [6]. There is some evidence that standard fecal indicators may originate from non-enteric sources, may survive significantly longer in tropical waters than in temperate ones, and may even become part of the aquatic microbial community [8]. *E*. *coli* thrives in the intestinal tract of humans and other warm-blooded animals and can contaminate water sources via untreated wastewater release [9,10]. The majority of *E*. *coli* strains are commensal; however, some lineages may have acquired specific virulence attributes that allow them to cause a wide spectrum of clinical manifestations, including diarrhea, urinary tract infections, meningitis, and septicemia [9,10]. It has been suggested that a full half of the total *E*. *coli* population resides in the external environment [11]. In this natural habitat, they can survive approximately 1 day in water, 1.5 days in sediment, and 3 days in soil [12]. This implies, in general, that the *E*. *coli* populations found in the secondary habitats are maintained by the constant arrival of microorganisms from hosts [11,13]. Some authors have suggested that *E*. *coli* and enterococci may multiply in warm, subtropical waters [12,14,15,16], and high concentrations of *E*. *coli* have been found in tropical surface waters in the absence of known human fecal sources [17,18]. *Salmonella* spp. are essentially vertebrate parasites, and in addition to humans, their natural hosts include all mammalian species, birds, reptilians and amphibians [19,20,21,22]. Consequently, in addition to polluted superficial water and groundwater, they may be present in various types of natural waters, such as rivers, lakes and estuaries [23]. Compared to other bacteria, *Salmonella* has higher survival rates in aquatic environments, ensuring its passage to the next host [24].

Other studies have demonstrated the extended persistence of indicator bacteria in the sediments of environmental waters [25,26] and in bromeliad water [27]. Anderson et al. [13] used laboratory microcosm experiments to show that the persistence tended to be greater in sediments than in fresh water columns and that there are differential survival rates for certain *E*. *coli* and *Enterobacter* strains in the two habitats. According to Winfield and Groisman [12], *Salmonella* spp. can survive in a septic tank for 10 to 15 days, can adhere to the substrata of a river, and may survive in soil for up to one year.

The Hyporheic Zone (HZ) is one of the most important interstitial freshwater habitats. It is a sub-benthic habitat composed of the spaces between the riverbed particles, thus representing a natural interface between stream water and subterranean water [28,29]. The exchange of water, nutrients, and biota between surface water and groundwater in the hyporheic zone has been demonstrated in various past publications [30,31,32,33,34,35]. This unique environment allows biological and chemical microzones to occur, facilitating diverse microbiological processes in a small volume and affecting the bacterial population dynamics.

In this zone, the microbiota consists of bacteria, archaea, protozoa and fungi [31]. These form thin films, called biofilms, on the granules. The biofilms are perhaps the most important component of the interstitial environment [36] and are estimated to spread over surfaces of up to 1 m^2^ in 10 grams of sediment with particle diameter of 0.1 mm [37].

The HZ is becoming increasingly recognized for its role as a nutrient contributor, providing a storage area for allochthonous and autochthonous organic matter. Its concentrations of nitrates, phosphates, silicates, organic carbon and heavy metals are higher than on the surface, and it is a habitat in which bacteria process the dissolved organic carbon more efficiently [38].

From the ecological point of view, the HZ and its biological components are strongly and persistently influenced by anthropogenic disturbances such as the discharge of industrial or municipal waste [39] and mining and deforestation [40]. The temperature, pH, inorganic nutrients, dissolved organic carbon and dissolved oxygen all have been found to affect the diversity, activity and metabolism of the microbial community in different HZ environments [33]. Recent studies on diversity have detected microorganisms such as fungi, heterotrophic aerobic bacteria, aerobic and anaerobic ammonium-oxidizing bacteria in the HZ [30,34,35]. Little information is available regarding the value of microbial indicators for fecal pollution in tropical regions [41]. Contamination of well waters by *Salmonella* spp. has been documented [42]. *Salmonella* spp. were detected in the HZ by Braioni et al. [43]. However, a significant knowledge gap exists in terms of the identities and roles of enteric bacteria in neotropical HZs. The aims of this study are: (1) to illustrate the results of the first survey of enteric bacteria in a neotropical stream, (2) to determine possible correlations between microbiological, physicochemicaland environmental parameters of the interstitial HZ, and (3) to define variables that influence this microbiota to lead future research in the neotropical region.

### Study Area, Sampling Characterization and Analysis

#### Study Area

Brazilian legislation does not require permission for sampling water, but our sampling permission reference is:Ministerio do Meio Ambiente-ICMbio-SISBIO n.32349-2 23/04/2013.

The Tijuca National Park is located entirely within the urban area of Rio de Janeiro, Brazil, between S22° 55’-S23° 00’ and W43° 11’-W43° 19’and contains an area of approximately 32 km^2^. Its vegetation is characteristic of the Atlantic Forest biome [44]. The climate is humid and subtropical, with an average annual temperature between 20°C and 25°C and an annual rainfall of more than 1,500 mm. The distribution of rainfall is characterized by two seasons: the rainy season, between November and February (with more than 250 mm/month of rain), and the dry season, from June to September (with less than 100 mm/month of rain). At other times of the year, the rainfall is within these limits. The geological substratum is predominantly comprised of granite [45].

**Fig 1:**
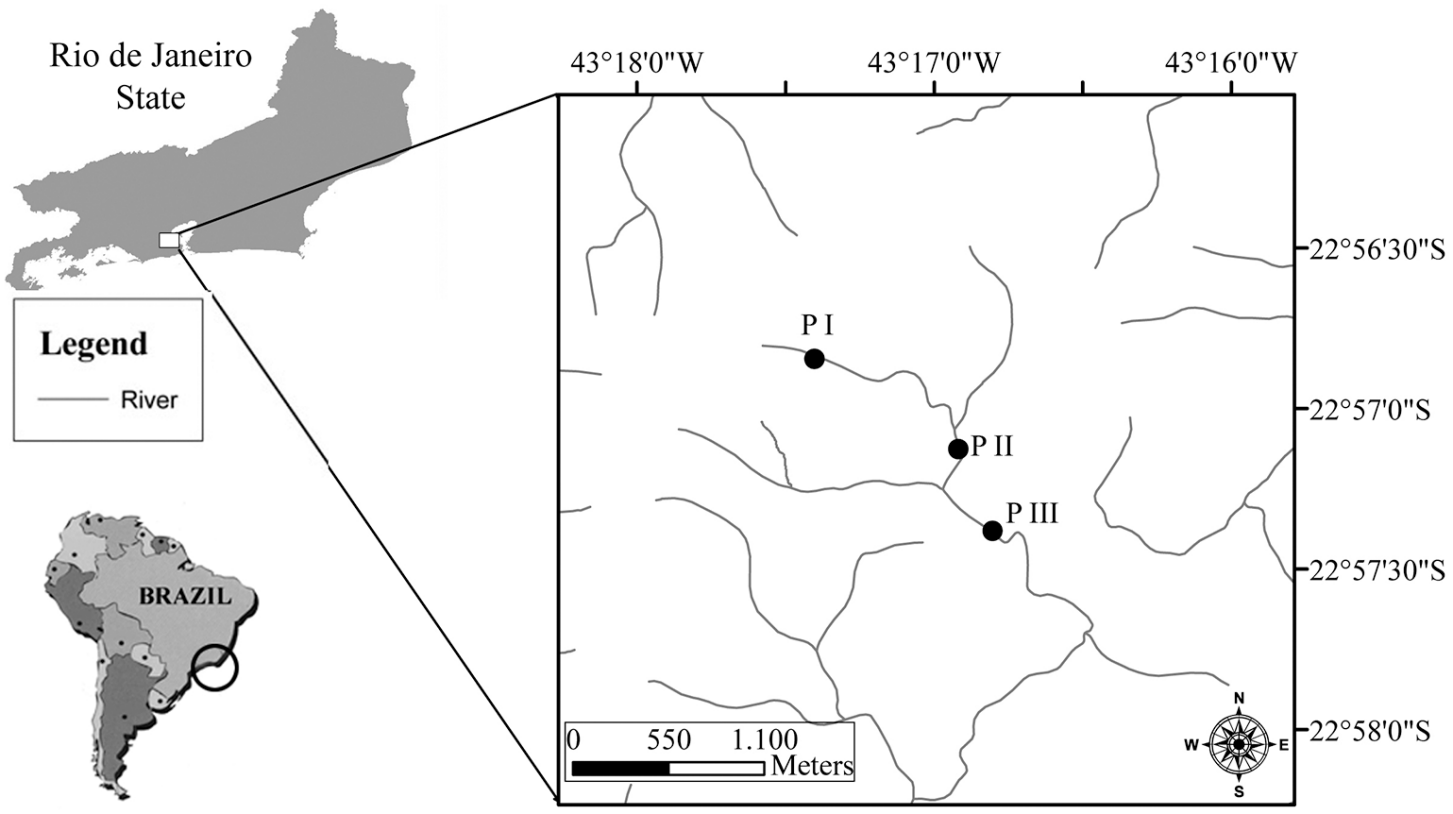
Map of the study area. Map of the study area showing the sampling sites in the Tijuca River (Tijuca National Park, Rio de Janeiro, Rio de Janeiro State, Brazil).

Samples were collected in October 2012 from three stream reaches (P I: 22°57'35.68"S, 43°16'33.54"W, 530 m altitude; P II: 22°57'13.08"S, 43°16'55.45"W, 475 m altitude; P III: Lat. 22°56'56.15"S, Long 43°17'9.84"O, 355 m altitude), located in the first, second and third orders, respectively (*sensu* Strahler [46]) in the Tijuca River (Fig 1).

To describe the sampling sites a visual-based habitat assessment system was used [47]. The habitat was defined valuing the structure of the surrounding physical habitat that influences the condition of the resident aquatic community as the variety and quality of the substrate, channel morphology, bank structure, and riparian vegetation.

The sampling sites used were quite different. In the first order site (P I), boulders covered 80% of the riverbed and the sand filled the cavities between the rocks; the transverse section of the riverbed was concave. The second order site (P II) was less steep, with sand representing 60% of the riverbed. The rest was comprised of cobble and coarse gravel, with regular riffles and pools; in this site, the transverse section of riverbed was flat. In the third order site (P III), the riverbed was 60% boulders, 30% pebbles, and 10% coarse sand. Here, the riffle-pool sequences were less regular and were separated by long areas of fine sediment deposition; the transverse section of the riverbed was concave. In this site, the stream received no treated effluents from the park administration and visitation center.

The Alfonso Viseu water-collecting checkpoint for CEDAE (the water company of Rio de Janeiro city) supplied street of the Alto da Tijuca neighborhood of Rio de Janeiro and was located three hundred meters downstream of the second order site.

### Sample collection

**Fig 2:**
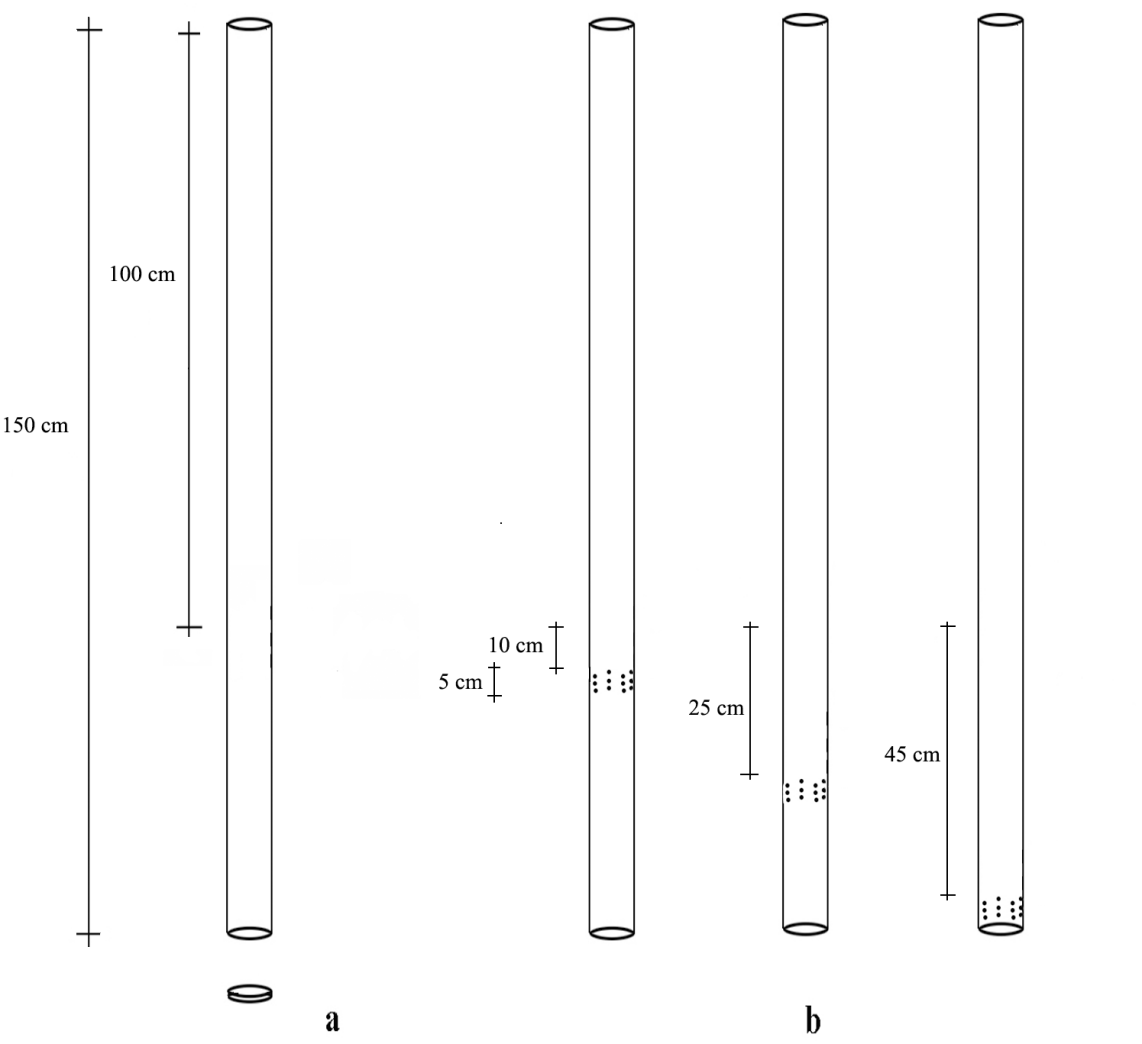
Mini-piezometers. Mini-piezometers and the position of the perforated bands at the different sampling depths.

At each site, polyvinyl chloride (PVC) mini-piezometers of 3/4" (2.6 cm) in diameter with holes of 0.5 cm in diameter distributed along a 5 cm perforated band were positioned in July 2012 at three depths: 10 cm (I); 25 cm (II) and 45 cm (III) (Fig 2).

No water was found in the sampling site located at a depth of 45 cm in the third order. For each depth, five mini-piezometers were positioned in four habitat typologies: up riffle (a), down riffle (b), lateral side of pools (c) areas of sand accumulation (bars, curves) (d1 and d2), for a total of 40 mini-piezometers.

Hyporheic and surface water samples of 0.5 liters each were collected in October 2012 using a diaphragm pump. However, four mini-piezometers were occluded, so a total of thirty-six samples were collected. All samples were stored and transported in a cool box kept below 4°C.

### Environmental parameters: hydraulics and limnology

In the field, the Vertical Hydraulic Gradient (VHG), Hydraulic Conductivity (K_H_), water suction time, oxygen, temperature and mini-piezometer depth were determined as described in Malard et al. [37].

The VHG describes the direction and intensity of the water exchange between the HZ and the surface or the subterranean zone. K_H_ is a measure of the ability of a porous material to allow fluids to pass through it. Oxygen and temperature were measured in the mini-piezometer using an oximeter.

In the laboratory, several physicochemical parameters were assessed. These included the Total Hardness, Calcium Hardness, Magnesium Hardness, Total Alkalinity, Hydroxide Alkalinity, Carbonate Alkalinity, Bicarbonate Alkalinity in mg/L CaCO_3_, mg/L Ca, mg/L Mg, pH, SO_4_^-^, Chloride (mg/L Cl^-^), Conductivity (μS/cm), mg/L OH^-^, mg/L CO^-^, mg/L HCO^-^, mg/L Total Dissolved Solids (TDS), mg/L salinity, mg/L N-Nitrogen, mg/L N-Nitrite, and Sulfate (mg/L SO_4_^-^). The alkalinity was determined via titration with indicators, while the total hardness and calcium hardness were measured by using titration with EDTA. The chloride parameters were determined with the Mohr titration method, the conductivity and TDS were determined by the electrometric method using a conductivity meter, and the Nitrogen ammonia was determined via the Nessler method. The sulfate levels were measured by the turbidimetric method, the Nitrogen nitrite levels by the diazotization method, and the pH by the potentiometric method, using a pH meter as recommended for the APHA [48], FEEMA [49] and FUNASA [50] methods. All analyses were completed in duplicate, and the results are expressed as means.

### Bacteriological Analysis

Hyporheic and surface water samples were collected in sterile bottles and transported under refrigeration for immediate laboratory processing. Water samples (5 mL) were diluted (10^-1^ to 10^-2^) in phosphate water (45 mL, solution A 0.25 M KH_2_PO_4_, solution B 0.4 M MgCl_2_.6H_2_O, pH 7.5) and filtered (10 mL) on a 0.45 μm cellulose acetate membrane (Millipore) [51]. The surface plate technique was used for the simultaneous detection of the total coliforms, *E*. *coli* and *Salmonella* spp. Duplicate plates were used for each dilution step. The membrane containing the retained bacterial cells was inoculated onto Chromocult coliform agar (CCA) for 18–24 h at 37°C [41]. After the bacterial growth period, colonies were assigned to bacterial species using their phenotypic traits, including morphological and physiological characteristics. Colonies between a salmon and red color were produced by salmon-galactoside cleavage by b-D-galactosidase, and these were classified the coliforms. In contrast, the dark blue to violet colonies resulted from salmon-galactoside and X-glucuronide cleavage by b-D-galactosidase and b-D-glucuronidase, and such colonies were classified as *E*. *coli*. Color-less colonies were instead classified as *Salmonella spp*. The results from the quantification of bacterial contamination were expressed as colony-forming units (CFU) per milliliter of water [52]. All tests were performed in duplicate.

### Statistical Analysis

For the statistical analysis, the data were log(x+1) transformed. To avoid collinearity between the variables, a Pearson’s correlation was calculated, excluding correlated variables pairs with r>0.6 or r<-0.6 [53]. To compare the physicochemical data between the sites, the micro-habitats and the depths, a Principal Component Analysis (PCA) was completed with a correlation matrix. ANOVA analysis was used to compare the bacteriological characteristics between the sites, micro-habitats and depths. Canonical Correspondence Analysis (CCA) elucidated the relationships between the biological assemblages and their environment. The ordinal variables, such as the river order and the micro-habitat, were treated quantitatively, with the coding 0, 1, 2, 3 and so on [54]. Tests were performed using GraphPad Prism 5.0 and Past packages.

## Results

The total coliform counts were detected in all samples. *E*. *coli* and *Salmonella* spp. were each detected in the surface of one site: *E*. *coli* was detected at a concentration of 0.1 CFU/ml in the third order site, and *Salmonella* spp. was detected at a concentration of 0.5 CFU/ml in the first order site. In the hyporheic samples, the number of positive samples varied (Table 1).

**Table 1:** Bacteriological analysis and percentages of positive results.

Depth	Totals Coliforms %	*Escherichia coli* %	*Salmonella* %
**surface**	100	33	33
**I**	100	17	92
**II**	100	29	86
**III**	100	40	80

**Fig 3:**
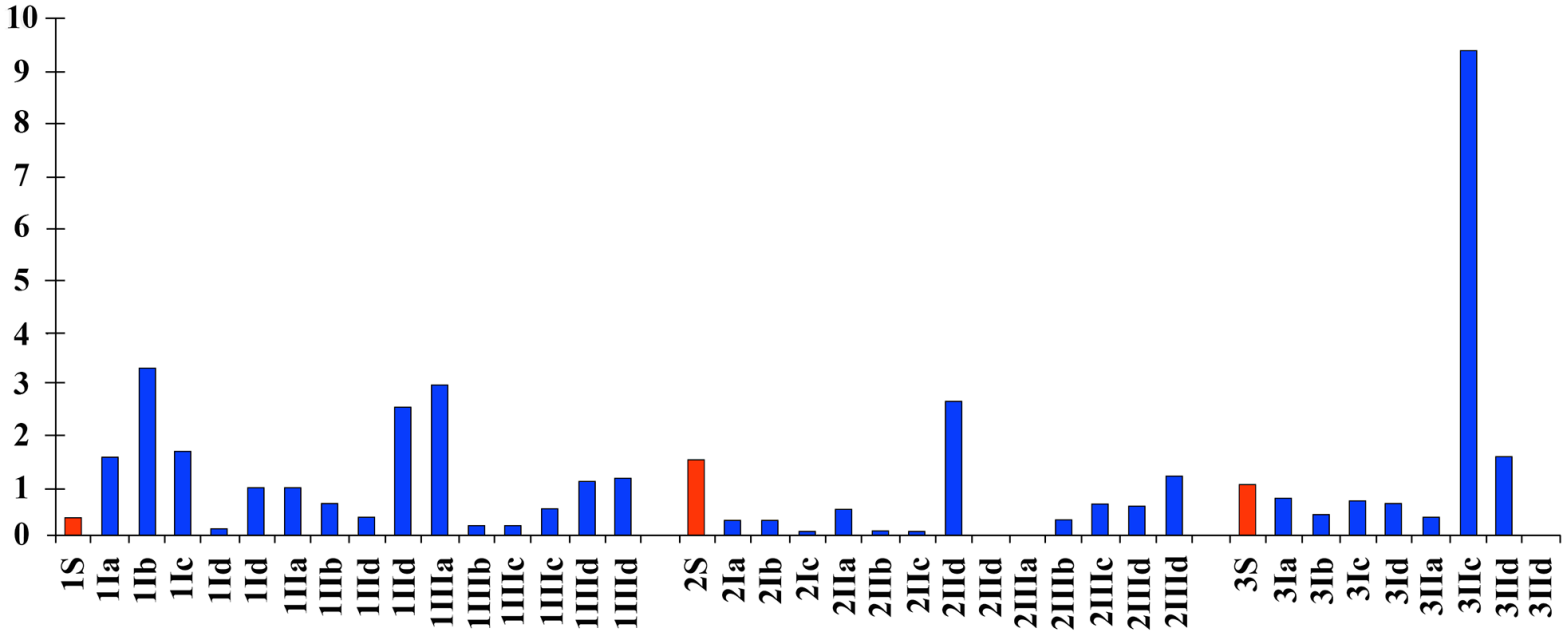
Total coliform. The total coliform levels (CFU /ml) at different sites, depths and habitat typologies. Surface samples are shown in red. Arabic numbers represent the sampling site. Roman numbers represent the sampling depth: I: 10 cm, II: -25 cm, III: -45 cm. Letters: a—up riffle, b—down riffles, c—lateral side of pools, d—areas of sand accumulation (bars, curves).

The maximum total coliform concentration (9.5 CFU) was found at a depth of 45 cm in the P III site on the wall of a pool. The highest values, between 2.5 and 3.5, for sites P I and P II were found at depths of 10 and 25 cm (Fig 3).

**Fig 4:**
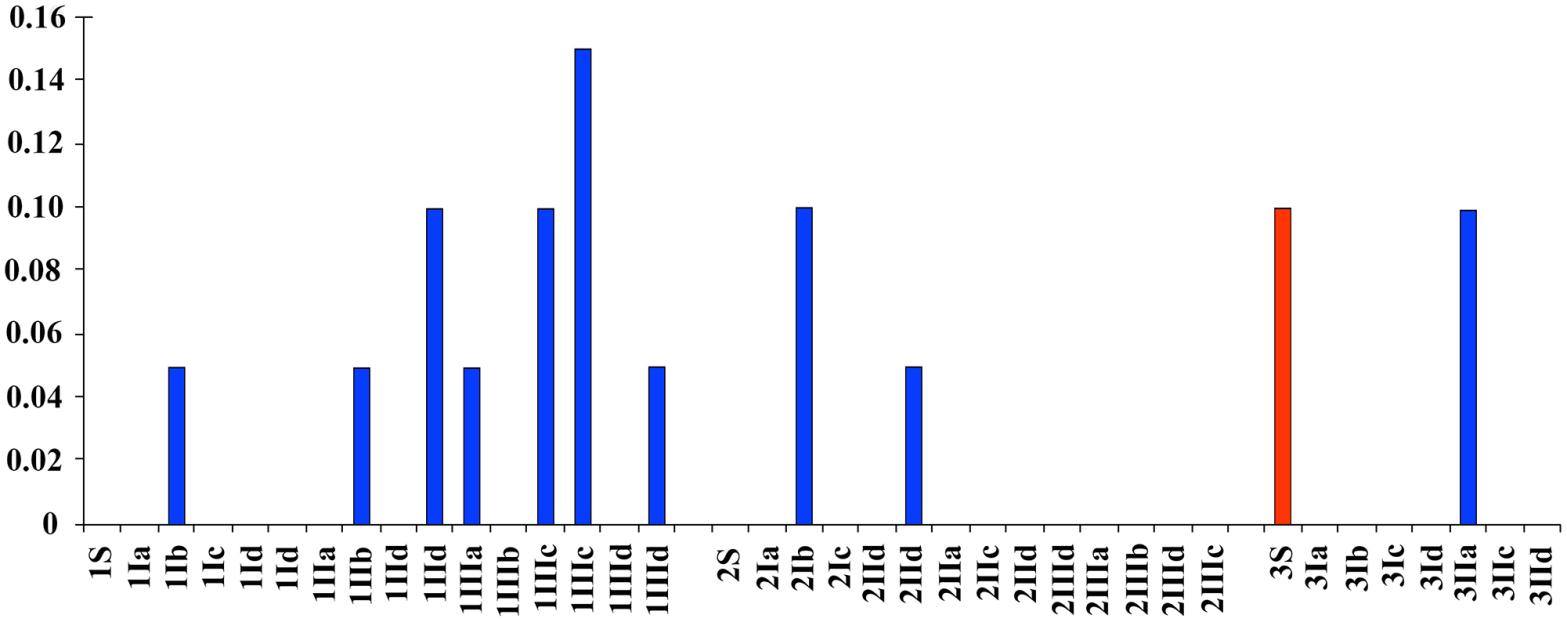
*Eschericia coli*. The concentrations of *E*. *coli* (CFU/ml) found at different sites, depths and habitat typologies. The surface samples are shown in red. Arabic numbers represent the sampling site. Roman numbers represent the sampling depth; the sampling depths were I—10 cm, II—25 cm, III -45 cm. The letters indicate the following: a—up riffle, b—down riffle, c—lateral side on pools, d—sand accumulation areas (bars, curves).

The maximum *E*. *coli* concentration (0.15 CFU) was found at the P I site at a depth of 45 cm on depth in a lateral side on pool. The highest value of sites P I, P II and P III at 10 and 25 cm depths was 0.1 CFU (Fig 4).

**Fig 5:**
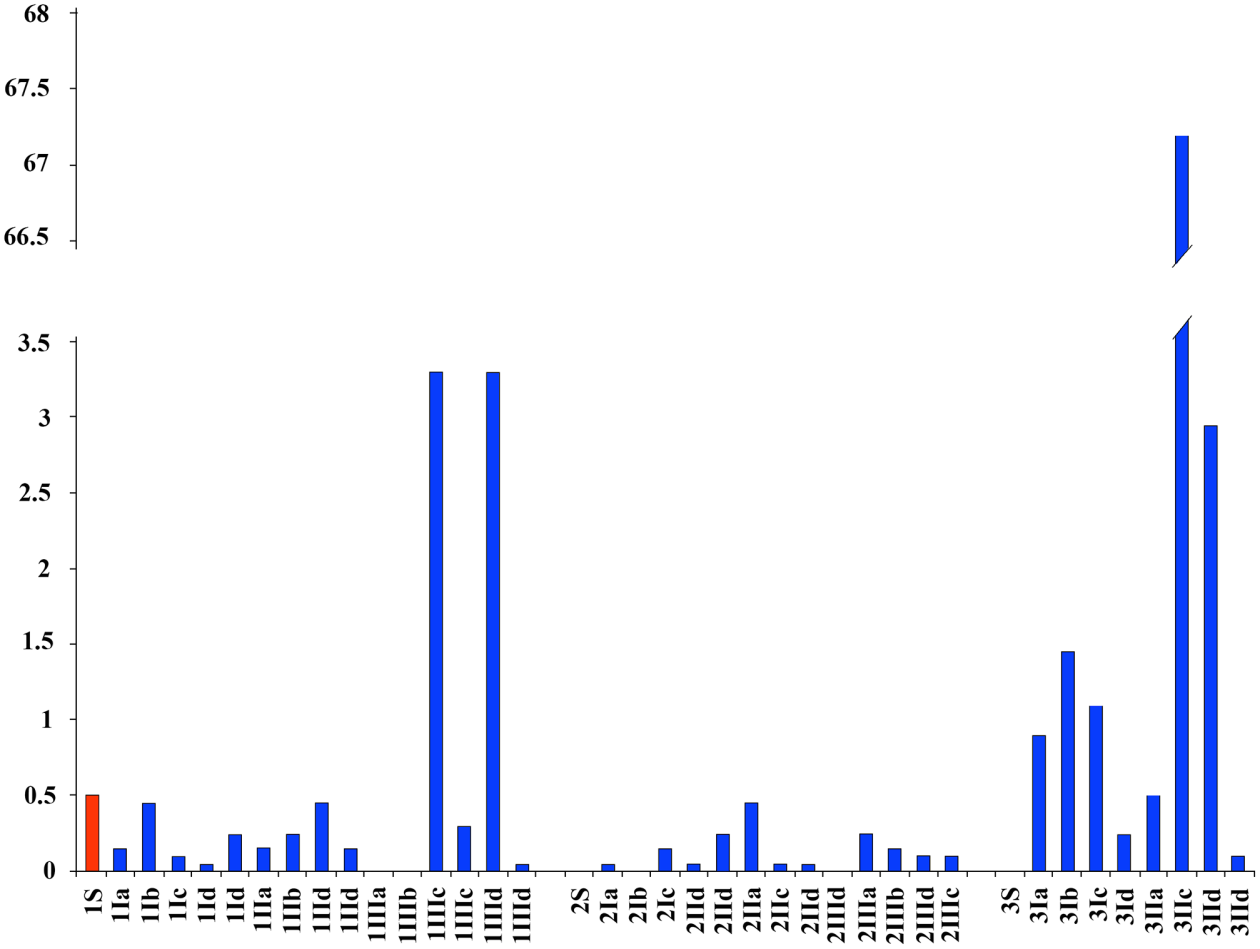
*Salmonella* spp. Amount of *Salmonella* spp. (CFU/ml) found at different sites, depths and habitat typologies. In red, surface samples. Arabic numbers represent the sampling site. Roman numbers represent the sampling depth: I, -10 cm; II, -25 cm; III, -45 cm. The letters represent: a—up riffle; b—down riffles; c—lateral side of pools; d—areas of sand accumulation (bars, curves).

The maximum concentration of *Salmonella* spp. (67.2 CFU) was found at site P III at a depth of 25 cm on lateral side on pools a pool. High values of 3.3 and 2.5 were found in the P I and P III sites at 45 and 25 cm depths lateral side on pools and in areas of sand accumulation (Fig 5).

The results of the physical and chemical analysis of surface and hyporheic samples are shown as mean values and standard deviations (Table 2). The most variable parameters were the conductivity and oxygen, while the least variable parameter was the pH; all decreased with increasing depth. From the physiochemical standpoint, the P II site is the most heterogeneous. In this sampling site, the calcium hardness and sulfate parameters were high and variable. In contrast, at PI and PIII, the temperature was lower and the oxygen and conductivity were higher, both varying with the minipiezometers.

**Table 2:** Physicochemical parameters.

Site	P I							P II							P III				
**depth**	SURF	I		II		II		SURF	I		II		III		SURF	I		II	
		mean	SD	mean	SD	mean	SD		mean	SD	mean	SD	mean	SD		mean	SD	mean	SD
**HardCa**	0.00	0.00	0.00	0.00	0.00	0.00	0.00	0.00	11.20	12.08	7.68	11.05	28.80	26.51	0.00	0.00	0.00	2.40	4.80
**pH**	5.79	5.92	0.08	5.65	0.20	5.61	0.18	5.69	5.17	0.36	5.41	0.31	4.92	0.11	6.17	5.79	0.24	5.77	0.03
**Sulfate**	4.02	4.65	1.20	6.36	0.98	7.01	4.50	2.93	27.26	31.14	17.63	17.92	53.52	43.59	4.39	8.20	7.27	4.16	0.89
**Chlor**	11.54	11.54	2.10	13.64	2.57	12.77	2.85	11.54	11.54	0.00	11.12	1.76	11.54	1.48	15.74	13.64	2.97	12.07	1.05
**Cond**	49.60	51.84	1.10	54.35	2.10	52.37	0.97	48.70	78.73	37.78	67.50	24.30	115.54	51.74	53.70	60.72	9.52	55.40	1.13
**Tot Alk**	14.72	9.20	1.84	10.12	3.52	8.59	3.00	11.04	7.37	0.02	7.74	2.01	8.10	3.08	11.04	9.57	2.94	9.21	2.11
**N-Nitrog**	0.00	0.00	0.00	0.02	0.03	0.00	0.00	0.01	0.00	0.00	0.01	0.03	0.01	0.02	0.00	0.01	0.02	0.00	0.00
**Nitrite**	0.00	0.01	0.00	0.01	0.01	0.01	0.00	0.00	0.00	0.00	0.01	0.01	0.09	0.16	0.00	0.01	0.01	0.00	0.00
**Ox%**	52.50	27.02	4.97	31.00	8.82	20.58	9.09	42.70	38.30	14.13	31.26	13.81	34.20	9.52	27.50	17.64	7.87	18.93	10.25
**Temp**	20.20	21.52	0.88	20.53	0.52	20.82	1.31	20.30	19.17	1.05	19.16	0.61	19.36	0.68	21.20	21.30	0.58	21.35	0.37

The physicochemical parameters of the surface and HZ, shown as the mean and standard deviation.

The P I, P II and P III, sampling sites were at I—10 cm, II—25 cm, and III—45 cm. SD, standard deviation; HardCa, calcium hardness; Chlor, chloride; Cond, conductivity; N-Nitrog, N-nitrogen; Temp, temperature.

A two-way ANOVA demonstrated significant physicochemical differences (P<0.0001, F 9, 49) the test was followed by a post tests two-way ANOVA using the Bonferroni method that show significant differences between P I and P II, as determined by the total hardness (P<0.01), calcium hardness (P<0.01), conductivity (P<0.001), TDS (P<0.001) and sulfate (P<0.001).

**Fig 6:**
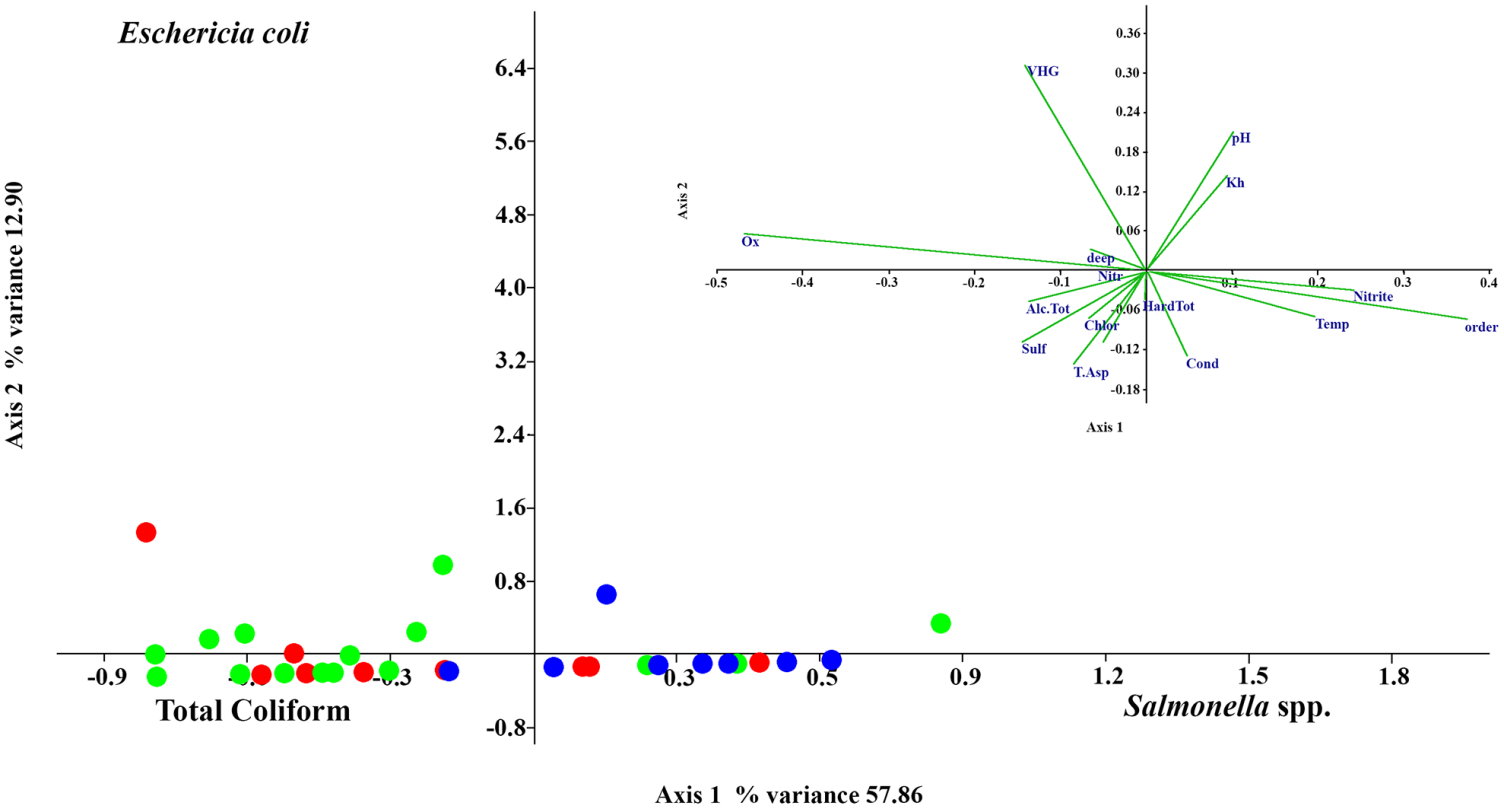
CCA analysis. CCA analysis with physicochemical, bacteriological and environmental data. The first order samples are shown in green, the second order samples are shown in red, and the third order samples are shown in blue.

The CCA analysis with bacteriological, physicochemical and environmental data (axis 1 eigenvalue 0.18679, % variance 57.86) discriminates *Salmonella* spp. from *E*. *coli* and the total coliforms (axis 2 0.039992, % variance 12.90) and discriminates the total coliforms from *Salmonella* spp. and *E*. *coli*. All three bacterial types were significantly influenced by the site, especially *Salmonella* spp. On axis 1, *Salmonella* spp. was found to be correlated with the oxygen level (-), river order (site) (+), nitrite (+), calcium hardness (-), temperature (+), sulfate (-) and VHG (-). In contrast, the total coliforms and *E*. *coli* levels were correlated with the same factor but in the opposite way. On axis 2, the total coliforms and *Salmonella* spp. are correlated with VHG (+), pH (+), K_H_ (+), suction time (-), Conductivity (-), Sulfate (-) and the micro-habitat (-). The *E*. *coli* levels were correlated with the same factors, but in the opposite way (Fig 6).

The same analyses were performed when excluding data from mini-piezometer 3IIc. The results showed no substantial changes.

**Fig 7:**
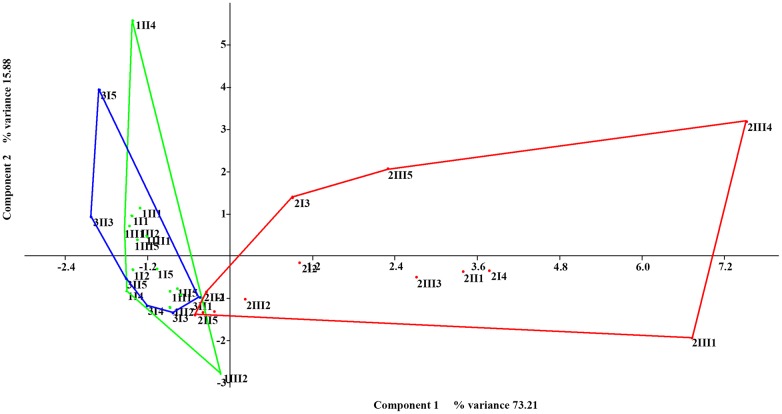
PCA with physicochemical data. The first order samples are shown in green, the second order samples are shown in red, and the third order samples are shown in blue.

The PCA analysis with the physicochemical data shows differences in P II relative to the other sampling sites (axis 1 eigenvalue 5.10935, % variance 73.21; axis 2 eigenvalue 2.76331, % variance 15.88) (Fig 7), especially in its calcium hardness (0.4202), conductivity (0.4161), STD (0.416), sulfate content (0.4086) and pH (-.3263). Of note, sulfates and Ca hardness could be detected only in this site.

**Fig 8:**
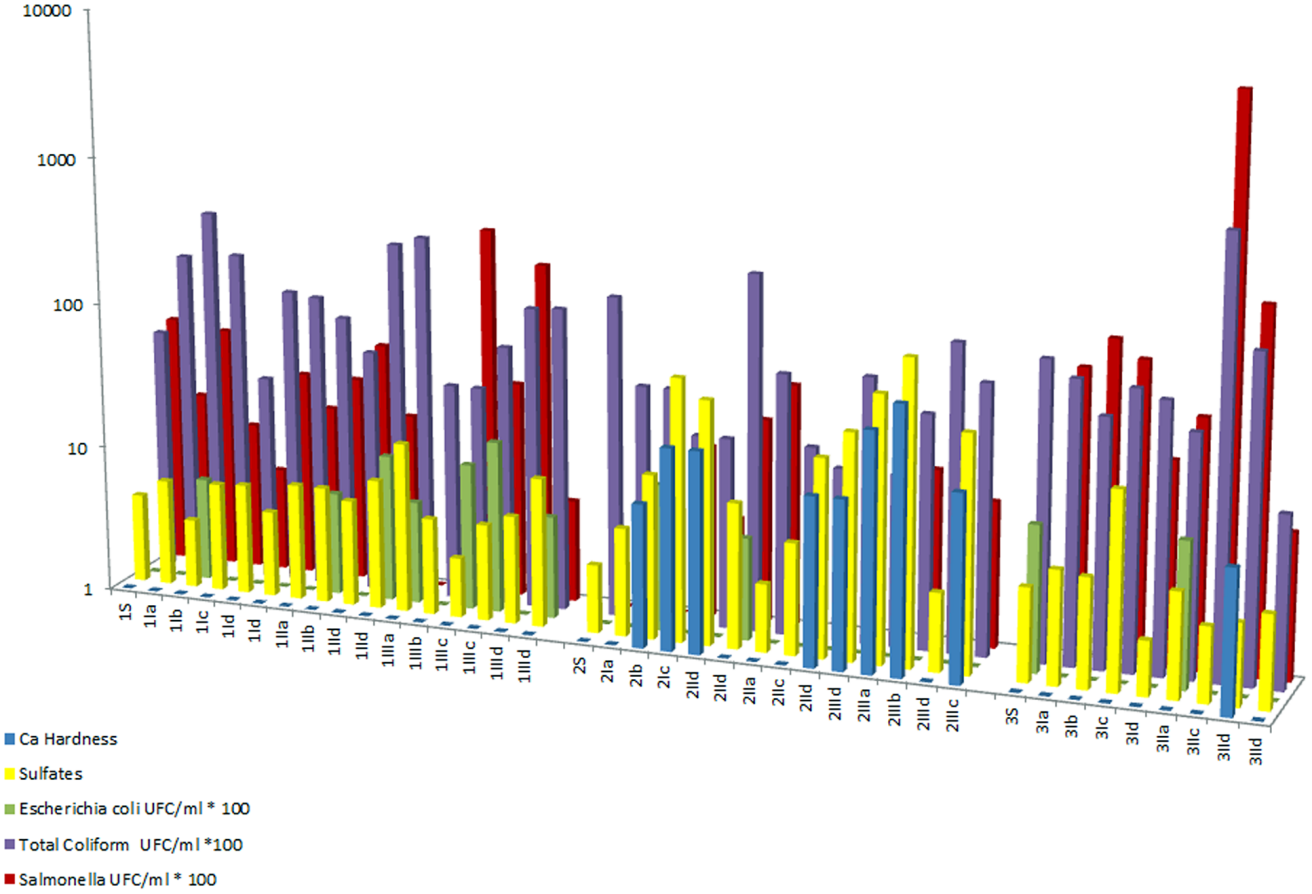
*Salmonella* spp., total coliforms, *Escherichia coli*, total hardness and sulfates. Histogram showing the *Salmonella* spp., total coliforms, *Escherichia coli*, total hardness and sulfates logarithmic scale for all measured sampling sites and depths.

The relationship between *Salmonella* spp., the total coliform count, *E*. *coli*, and the total hardness and sulfate concentration is shown in a histogram (Fig 8).

## Discussion

Since 1914, coliform counts have been used as a bioindicator of the microbiological quality of treated and untreated drinking water and as a standard indicator of recent fecal contamination in most countries.

Historically, many countries have been using fecal indicator bacteria as a monitoring tool to predict the presence of microorganims originating fromin fecal contamination. *E*. *coli* is still the bacteria that best satisfies the fecal indicator microorganism criteria since it is a species that is specific to the intestines of humans and other warm-blooded animals but is not necessarily pathogenic. Total coliforms include bacterial species of fecal origin, as well as other bacterial groups that commonly occur in the soil. Moreover, the persistence in water, the relationship between fecal indicator bacteria and the occurrence of diseases may not be accurate in all the locations of sampling and environmental settings [9,10,55,56]. Additionally, indicators may be severely underrepresented when injured by disinfectants used in water treatment processes.

The persistence and survival of these bacteria in underground media have been shown to be significantly influenced by a complex array of physical, chemical, and biological factors, including the concentration of indigenous microorganisms, the growth and decay rates, the concentration of available nutrients and the consumption kinetics [13, 57].

Hendricks and Morrison [58] showed that enteric bacteria could multiply in both polluted and unpolluted river water at temperatures as low as 10°C. Hendricks [59] and Burton et al. [60] reported an increased recovery rate of streambed sediment in relation to surface waters due to a combination of sedimentation and sorption, which provides protection from bacteriophages and microbial toxicants. The adhesion of *Salmonella* spp. cells to soil particles is correlated with cell surface hydrophobicity [61]. Bacterial association with soil particles provides bacteria with high concentrations of nutrients due to the release of organic molecules from attached algal cells. The association also gives the bacteria protection against predation, by providing shelter against protozoan grazing [25]. The long-term survival of Salmonella in water, manure, soil and sediment has been well documented [12], and a high quantity of coliforms have been recorded in unpolluted tropical waters [13,18,62]. As in the mammalian host environment, nutrients in some tropical ecosystems are maintained at high concentrations. With consistently warm air, soil, and water, they can provide an ideal habitat for the survival, growth, and proliferation of *E*. *coli* [13,18,62].

The prevalence of *Salmonella* spp. in the water is highly influenced by conductivity, temperature, alkalinity and oxygen concentration [63,64]. Jimenez et al. [17], Carrillo et al. [18] and Burton et al. [60] have all reported a strong positive correlation with the water temperature and nutrient concentrations and *E*. *coli* cells found in superficial water located upstream of known fecal sources in tropical rain forest ecosystems. Accordingly, our data identify the same parameters as being important determinants but also point to other key factors, such as the structure and composition of meso and microhabitat.

In the P III sampling site located in the third order at a depth of 45 cm, no water was found. This may be due to the presence of a cascade followed by a small upstream dam of 1.5 m in height. Dams along small-order streams are known to alter the link between the headwater and the downstream areas, potentially increasing the hyporheic connectivity in the riparian zone over short distances [65]. However, dams can also simplify the alluvial system structure, reducing peak flows, preventing rejuvenation of alluvial aquifer structure and reducing the hyporheic flow [66].

The P I and P III sampling sites were quite similar and were distinct from the P II site of second Straler’s river order in sand/silt quantity, heterogeneity, concavity, width and steepness, resembling a higher Straler’s order in structure.

The HZ can be an important sink or source for the stream ecosystem and is an important site for the remineralization of organic matter [38]. The organic matter found in the hyporheic sediments originates from surface carried by the downward flow of stream water [67,68] and from decaying organic particles, dissolved molecules, free-living organisms and attached biofilms [69,70].

Anderson et al. [71] and White [72] reported that the magnitude of vertical hydraulic gradients varies along the longitudinal stream profile. The hyporheic exchange flow increases predictably with increases in the water surface concavity, heterogeneity and channel unit spacing in headwater and mid-order streams [71]. Downwelling occurs when the stream profile is convex, when permeability increases in the downstream direction, or when the bed depth increases in the downstream direction. Conversely, upwelling occurs when the stream profile is concave, when permeability decreases in the downstream direction, or when the bed depth decreases in the downstream direction. The downwelling and upwelling zones differ significantly in terms of the temperature, pH, redox potential, and the dissolved oxygen and nitrate levels, with significant positive correlations occurring among the latter three factors. This creates distinct patterns in the microbial activity [73,74].

Sulfur is one of the major constituents of ground water, commonly occurring as sulfate molecules. In anoxic conditions, biofilms can use calcium for respiration [38]. These facts justify the measurements of Ca hardness and sulfate levels in the P2 sampling site. Calcium can be derived from the dissolution of plagioclase and/or fluorite minerals from volcanic and metamorphic stones; sulfates are generally of organic origin in anoxic environments and arrive via the upwelling process.

This work show that viable enterobacteria are present and probably reproduce in the HZ. Our results demonstrate that enteric bacteria are heterogeneously distributed along the sampling sites assessed. Their spatial variation was related to environmental factors and physical-chemical parameters within the HZ and may play a key role in the microbial diversity and distribution within these ecosystems. These factors possibly influence the survival and viability of bacteria. Our results revealed that *Salmonella* spp. and coliforms have quite different microhabitat preferences. *Salmonella* spp. and coliforms both are frequently deposited on the lateral sides of pools and curves and bars, but the two tend to distribute into different depths, probably due to the temperature. However, *Salmonella* spp. prefers compact substrata with a lower fluid flow and upwelling areas with less oxygen inflow. In contrast, coliforms have the opposite preference. Statistical analyses suggest the existence of different habitat preferences between the total coliforms and *E*. *coli*, but our data do not permit us to discriminate. The recovery of enteric isolates from particular HZ microzones exhibiting extreme physiological conditions was unexpected. These results strongly support the hypothesis that environmental enteric isolates may exhibit an important adaptive advantage capable of surviving and proliferating in this ecosystem. The circulation and persistence of these microorganisms in the HZ may promote close interaction and horizontal genetic exchanges. Considering that *E*. *coli* and *Salmonella* isolates may comprise virulent strains, the recovery of these microorganisms from the HZ water samples have implications for human health and disease risk. Virulence genetic markers are commonly encoded on mobile genetic elements and promote the rapid evolution of bacteria and the emergence of variants within species, including, pathogenic lineages.

Our data revealed the presence of viable enterobacteria in the HZ of a neotropical stream in Rio de Janeiro, Brazil, whose distribution differed significantly across microzones. This is of special relevance considering that circulating bacteria may comprise virulent strains or reservoirs of resistance determinants. The presence of potentially pathogenic bacteria in HZ raises questions about the increased risk to ecosystems and human health. Future work is needed in order to investigate molecular markers to better clarify the diversity, pathogenic potential and the biology of these circulating microorganisms as well as the population dynamics in this aquatic ecosystem. Similar to our work, a prior study, using metagenomic and amplicon sequencing, showed how environmental parameters can alter river sediment microbial community structure and function [75].

## Conclusions

The present study detected a wide occurrence of enterobacteria in HZ microzones of Tijuca National Park which spatial location leads to the hypothesis that these microorganisms may be sensitive to environmental parameters. The detection of *E*.*coli* and Salmonela in HZ does not guarantee that circulating microorganisms are pathogenic or resistant, thus requiring additional characterization target to specific virulence traits and antibiotic resistance markers. This may represent a safety risk, in view of the usage of this area’s water for human consumption and recreational activities.

Therefore, we must reevaluate the use of these bacteria as indicators of human-caused pollution in tropical regions. Further studies are needed to define the precise conditions and microhabitats necessary to determine the life cycle of these organisms in the HZ environment to prevent future health hazards.
